# Arterial Spin Labeling Perfusion in Pediatric Brain Tumors: A Review of Techniques, Quality Control, and Quantification

**DOI:** 10.3390/cancers14194734

**Published:** 2022-09-28

**Authors:** Abir Troudi, Fatima Tensaouti, Eloise Baudou, Patrice Péran, Anne Laprie

**Affiliations:** 1 Toulouse Neuro Imaging Center (ToNIC), INSERM-University of Toulouse Paul Sebatier, 31300 Toulouse, France; 2 Radiation Oncology Department, Claudius Regaud Institute, Toulouse University Cancer Institute-Oncopole, 31300 Toulouse, France; 3 Pediatric Neurology Department, Children’s Hospital, Toulouse University Hospital, 31300 Toulouse, France

**Keywords:** perfusion MRI, arterial spin labeling, cerebral blood flow, quality control, pediatric tumor

## Abstract

**Simple Summary:**

Pediatric brain tumors are the second most common type of childhood cancer and the leading cause of cancer death in children. Many survivors have long-term cognitive impairments and behavioral issues as a result of treatment, which may reduce their quality of life. Magnetic resonance imaging (MRI) techniques have revealed structural and functional brain changes that may be related to clinically observed cognitive impairment. A special emphasis has been placed on arterial spin labeling (ASL) MRI, which can be used to estimate changes in capillary perfusion after radiotherapy, in order to assess treatment response and neurocognitive sequelae. The current review was conducted to knowledge of ASL techniques and describe the main findings of research on the use of ASL in both healthy pediatric populations and patients treated for brain tumors.

**Abstract:**

Arterial spin labeling (ASL) is a magnetic resonance imaging (MRI) technique for measuring cerebral blood flow (CBF). This noninvasive technique has added a new dimension to the study of several pediatric tumors before, during, and after treatment, be it surgery, radiotherapy, or chemotherapy. However, ASL has three drawbacks, namely, a low signal-to-noise-ratio, a minimum acquisition time of 3 min, and limited spatial summarize current resolution. This technique requires quality control before ASL-CBF maps can be extracted and before any clinical investigations can be conducted. In this review, we describe ASL perfusion principles and techniques, summarize the most recent advances in CBF quantification, report technical advances in ASL (resting-state fMRI ASL, BOLD fMRI coupled with ASL), set out guidelines for ASL quality control, and describe studies related to ASL-CBF perfusion and qualitative and semi-quantitative ASL weighted-map quantification, in healthy children and those with pediatric brain tumors.

## 1. Introduction

The delivery of oxygenated blood and nutrients to brain tissue is known as brain perfusion. It is primarily studied at the level of microcirculation, which occurs in the blood system’s smallest vessels, (i.e., capillaries). Nuclear imaging, computed tomography (CT), positron emission tomography (PET), single photon emission computed tomography (SPECT), and magnetic resonance imaging (MRI) are all medical imaging modalities that can be used to assess perfusion [1,2].

Originally developed as an MR angiography method [3], arterial spin labeling (ASL) is one of the techniques employed to measure brain perfusion. Water is used as a freely diffusible tracer.

ASL has emerged as a promising alternative to nuclear imaging and CT for assessing tumor vascularity, as it does not require the injection of an exogenous contrast agent. It is particularly relevant for the imaging of pediatric populations, as it obviates the need for the irradiation of a PET scanner, the laying of a venous line, and the injection of gadolinium or a T2 perfusion sequence. Studies using ASL have shown promising results for the assessment of malignancy in pediatric brain tumors [4,5,6].

The primary advantages of ASL over other modalities are thus its non-invasiveness, which allows for the study of brain perfusion in healthy individuals, and its ability to investigate cerebral blood flow (CBF) and brain metabolism (resting state ASL) [7]. It is also becoming more popular in the study of functional brain connectivity development; as it does not require the use of external tasks, which is more convenient than performing task activation functional MRI (fMRI) in infants and younger children. Furthermore, its quantification of perfusion parameters allows for patient-specific studies such as tumor explorations [8] and tumor grading [9].

ASL could be used to measure responses to treatment, as well as the neurocognitive deficits in executive functions, verbal processing, attention, and memory that are often observed in long-term survivors of pediatric brain tumors. It could even prevent or reduce neurocognitive dysfunction by quantifying perfusion according to variations in irradiation, one of the main contributors to neurocognitive sequelae [10,11].

Before conducting any quantitative measurements, it is very important to consider other perfusion modifiers besides medication that might influence measures of perfusion in the human brain [12].

Various parameters for characterizing this perfusion have been identified in the literature, and under certain assumptions, ASL is capable of quantifying several of these. CBF, which is usually expressed in milliliters of blood per minute per 100 g of brain tissue, is the most commonly mentioned parameter in ASL [13].

CBF quantification by ASL is reliable, reproducible, and correlates with other imaging modalities, particularly nuclear medicine techniques. ASL-CBF can therefore reveal perfusion abnormalities, especially brain diseases, in a completely non-invasive way.

This technique nevertheless has a number of limitations. For a start, ASL sequences are often performed as part of a complex and complete MRI work-up, with a long acquisition protocol, thereby accentuating the risk of artifacts. Other limitations include a low signal-to-noise ratio (SNR), limited spatial resolution, and a short label lifetime [14].

Image-quality assessment is therefore a fundamental step before making any clinical judgment based on ASL perfusion MRI [15]. Investigating brain perfusion is of interest both in healthy children [16] and in patients with pediatric tumors such as medulloblastomas [11], low-grade gliomas [17], glioblastomas [18], pilocytic astrocytoma [19], and ependymomas [20]. It allows perfusion and CBF changes in pediatric brain tumors to be quantified before, during, and after surgery, radiotherapy, and chemotherapy.

The goal of the present review was to provide a comprehensive update of ASL perfusion techniques, under the following seven headings: (1) ASL perfusion principles and methods, (2) CBF quantification, (3) technical advances in ASL, (4) quality control of various types of artifacts and negative values in ASL-CBF maps, (5) CBF perfusion quantification in healthy children and patients with pediatric brain tumors before and after treatment, (6) ASL perfusion-weighted map evaluation, and (7) summary.

## 2. ASL Perfusion Principles and Methods

### 2.1. Principles

**Figure 1:**
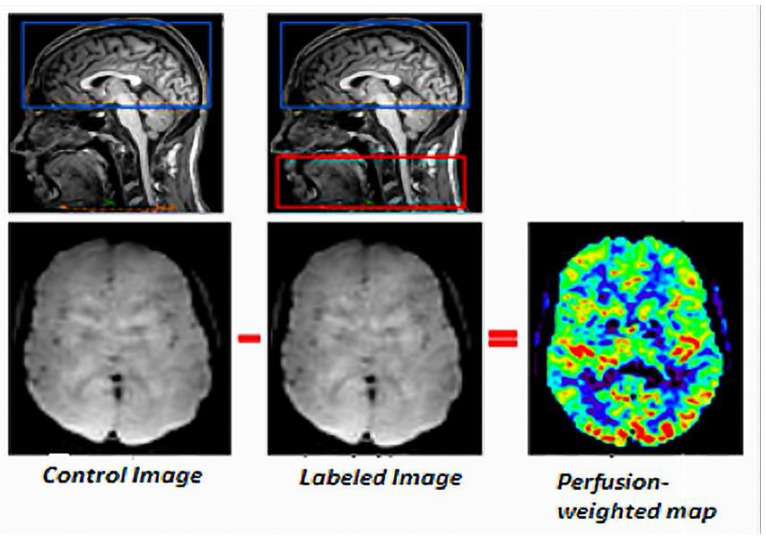
General principle of ASL.

The first experimental ASL method was devised by Williams et al. [3]. Their experiment was conducted on rats at a field intensity of 4.7 T, to measure the endogenous perfusion signal coming from arterial blood by manipulating its magnetization [21]. ASL is performed non-invasively in an ultra-rapid-echo-planar type sequence by acquiring two consecutive images: one with labeled arterial protons and one without. The Figure 1 depicts the general concept of the ASL technique [22].

Magnetic labeling of arterial proton spins is achieved by applying one or more radiofrequency (RF) pulses upstream of the volume of interest, altering the measurable magnetization and T1 relaxation time. These RF pulses are used to reverse the longitudinal magnetization of arterial blood at the neck vessels [23]. The labeled spins then migrate through the arterial network to the brain, where they perfuse brain tissue and produce an image of the brain. This acquisition is made at an inversion time (TI) that represents the amount of time it takes for the spins to pass from the capillary compartment to the extravascular one. The second image is captured in control mode (i.e., without spin labeling). Subtracting the labeled image from the control image yields the perfusion image [24]. This image, which only represents 0.5–1.5% of the total signal, provides information about the amount of marked arterial magnetization. Many factors influence it, including CBF, tissue relaxation time, and inversion time. When a weak signal is obtained, many repetitions are frequently required to ensure a sufficient SNR [23].

### 2.2. Techniques

There are four main ASL techniques: continuous (CASL), pulsed (PASL), pseudo-continuous (pCASL), and velocity selective (VSASL).

#### 2.2.1. Continuous Arterial Spin Labeling

**Figure 2:**
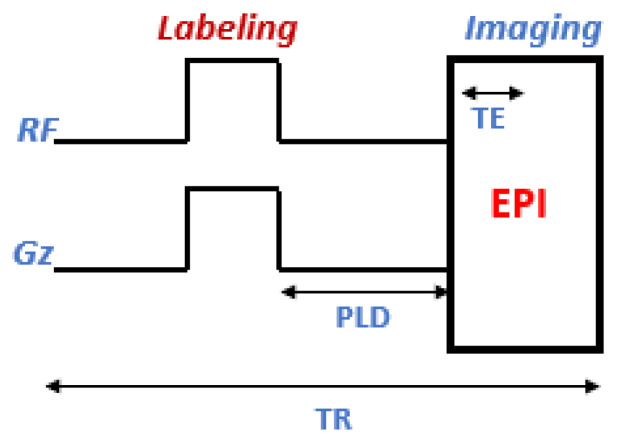
Schematic representation of the CASL sequence. EPI: echo planar imaging module; TE: echo time; PLD: post labeling delay; TR: repetition time; G_z_: gradient.

It was Williams et al. who first proposed CASL in 1992 [25]. It is carried out via a thin slice at the neck level. Magnetization reversal is accomplished by adiabatically reversing the spins of arterial water with a continuous 2–4 s RF pulse combined with a gradient pulse in the direction of the flow (Figure 2). In general, CASL allows for more robust labeling of arterial magnetization and thus provides greater perfusion contrast than other types of labeling [26]. The effect of magnetic transfer (MT) and the high level of energy deposited in the tissue (specific absorption rate, SAR) are the two major limitations of this technique.

The MT effects are caused by partial saturation of the macromolecules and a reduction in the water signal in the volume being studied. Several methods have been devised to reduce these effects, such as beginning with single-slice acquisitions and progressing to multi-slice ones. This approach is based on the use of additional RF pulses during control acquisition, but at the expense of increased SAR [27]. The latter can be avoided by using other antennas for marking the spin besides the image acquisition coil [28].

#### 2.2.2. Pulsed Arterial Spin Labeling

**Figure 3:**
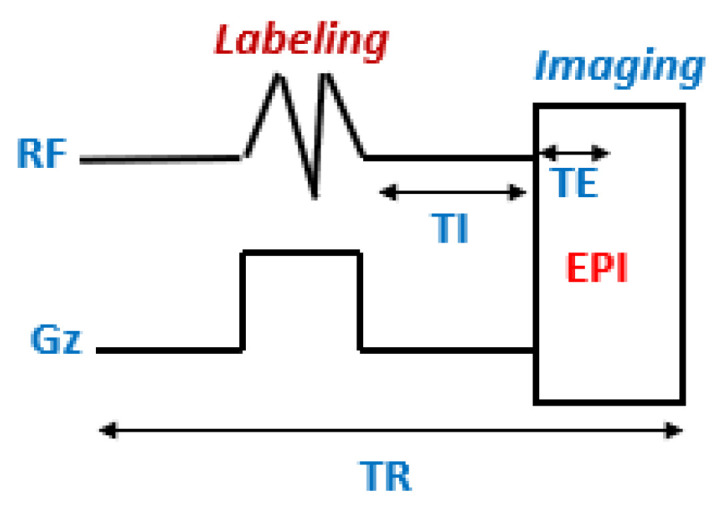
Schematic representation of the PASL sequence. EPI: echo planar imaging module; TE: echo time; TI: inversion time; TR: repetition time; G_z_: gradient.

PASL is a technique that uses very short RF pulses to perform spin labeling over a large labeling area (Figure 3) [29]. PASL sequences can be divided into two types according to the labeling area: symmetrical [30], when the labeling plan is applied symmetrically to the imaging volume, and asymmetrical, when the labeling area is located upstream of the volume of interest.

#### 2.2.3. Pseudo-Continuous Arterial Spin Labeling

**Figure 4:**
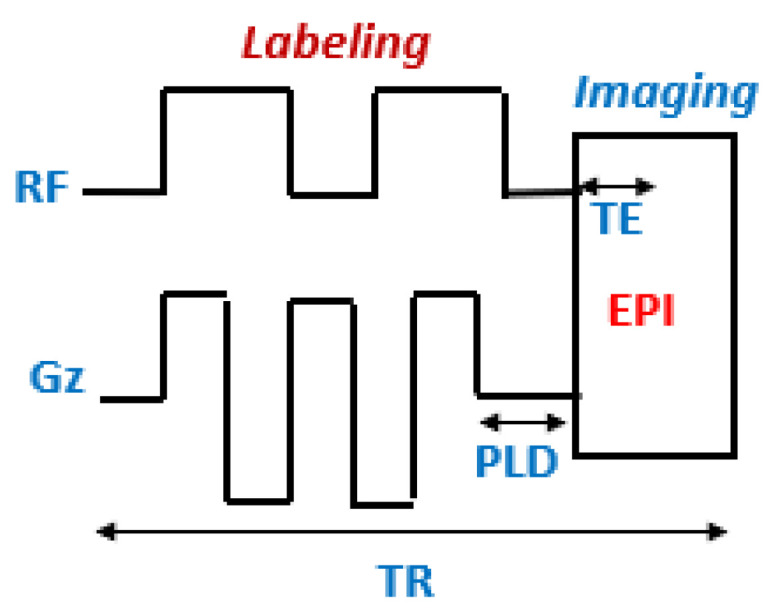
Schematic representation of the pCASL sequence. EPI: echo planar imaging module; TE: echo time; PLD: post-labeling delay; TR: repetition time; G_z_: gradient.

To limit the duration of the application of the radio frequency, CASL can be improved via the use of several RF pulses (Figure 4), via the body antenna. This method, known as “pseudo-continuous ASL” (pCASL), combines the advantages of CASL and PASL, but not their disadvantages, which explains its rapid development in clinical research [15,31].

#### 2.2.4. Velocity Selective Arterial Spin Labeling

VSASL was developed to overcome the limitations of spatially selective ASL (CASL, PASL, pCASL). It is applied to the acquired volume itself and is not limited by spatial location, so it reduces and/or eliminates arterial transit time (ATT)-related signal loss.

In VSASL, a short train of RF and flow-sensitive gradient pulses labels arterial blood that moves faster than a prearranged velocity, known as the cutoff velocity (Vcut). During imaging, spins issuing from the same Vcut are saturated; then, the labeled arterial blood decelerating below the Vcut is measured. As a result, a higher SNR was provided by an achieved ATT = 0 across the entire imaging volume, and a used 0 PLD to minimize ASL signal decay due to T1 relaxation.

**Figure 5:**
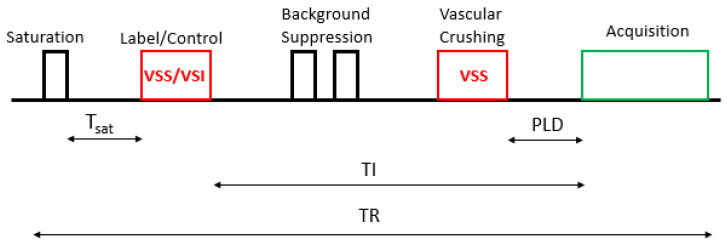
Schematic representation of the VSASL sequence. PLD: post-labeling delay; TI: inversion time; TR: repetition time.

This method is based on the pulse diagram sequence shown in Figure 5. The arterial spins are labeled by the VS labeling (label/control) module (VS saturation (VSS)/VS inversion (VSI)) after a saturation time (Tsat). A vascular crushing module (VCM) is then applied, with the same Vcut for acquiring as for labeling, to define the temporal width of the VSASL bolus [32].

## 3. CBF Quantification

The labeled images are subtracted from the control ones, yielding perfusion-weighted images. An additional proton density S(PD) image must be combined with the different images to calculate CBF.

The quantification of CBF is available in the ASL technique for all three types of labeling: PASL, CASL or pCASL, and VSASL.

Buxton et al. [33] created the general quantification model in ASL in 1998. This model expresses the difference in magnetization between the control image and the labeled image (M(t)) as the sum of three components (transit time effects of labeled spins between labeling region and region of interest (ROI), blood-tissue exchanges, and relaxation times):

∆*M(t)* = 2*M*_0*b*_*·f·∫·c(t′) r(t − t′) m(t − t′) dt′* (1)

where *M*_0*b*_ represents the longitudinal magnetization of blood protons at equilibrium, *c(t^’^)* the fractional arterial input function, *r(t − t′)* the labeled proton output of the voxel, and *m(t − t′)* longitudinal relaxation effects. This model applies to CASL and PASL, subject to adapting the parameters. However, there are many sources of quantification errors: transit time, vascular artifacts, shape and efficiency of the inversion pulse, bolus dispersion effect, blood-tissue distribution coefficient of labeled protons, and magnetization of blood at equilibrium [34].

For the quantification of *CBF* in each voxel, a relatively basic model has been proposed for each type of labeling [35,36]:

- CASL/pCASL
  (2) $$CBF=\frac{6000\;\lambda \;\left(SI_{conrol}-SI_{label}\right)e^{\frac{TI}{T1_{blood}}}}{2\;\alpha \;TI_{blood}\;SI\left(PD\right)\left(1-e^{\frac{-\tau }{T1_{blood}}}\right)}$$
- PASL
  (3) $$CBF=\frac{6000\;\lambda \;\left(SI_{conrol}-SI_{label}\right)e^{\frac{TI}{T1_{blood}}}}{2\;\alpha \;TI_{1}\;SI\left(PD\right)}$$
  where *λ* is the brain/blood partition coefficient in mL/g, *SI_control_* and *SI_label_* are the time-averaged signal intensities for the control and labeled images, *T1_blood_* (ms) is the longitudinal blood relaxation time, *α* is labeling efficiency, *SI(PD)* is the signal intensity of PD images, and *τ* (ms) is the labeling duration. *PLD* (ms) is the delay after labeling, *TI* (ms) is the bolus duration in PASL (i.e., equivalent of labeling duration in CASL/pCASL), and *TI_1_* (ms) is the reversal time in PASL. The factor 6000 converts the mL/g/s unit to mL/100 g/min, as described in the literature.
- VSASL
  (4) $$CBF=\frac{6000\;\lambda \;\Delta S\;e^{\frac{TI}{T1_{blood}\;}}\;\left(1-e^{\frac{-T_{sat}}{T1_{blood}\;}}\right)}{2\;\alpha \;\tau \;SI\left(PD\right)}$$
  where *λ* is the blood-tissue partition coefficient, ∆*S* the signal difference between the label and the control images, *τ* (ms) the time between labeling, and *TI* (ms) the time between labeling pulse and readout. *T1_blood_* (ms) is the T1 value of the blood. *SI(PD)* is acquired PD and *Tsat* (ms) the saturation.

In cases where the *SI(PD)* acquisition is not available, an estimation of PD must be made to quantify the CBF. This requires several conditions to be met, as described by Pinto et al. [37]. These authors investigated the effects of various post-processing options in ASL data calibration on perfusion quantification and reproducibility. There are three main strategies for creating *SI(PD)* maps:

- Long TR calibration scan: This method is based on a separately acquired long TR scan that approximates the tissue’s equilibrium magnetization in each voxel.
- ASL control averaging: If no background suppression is used, the *SI(PD)* map can be estimated by averaging the control images at a fixed TI, which should then be corrected for the amount of T1 relaxation during TI at each voxel, to yield a corrected map of the tissue equilibrium magnetization.
- Control saturation recovery: If no background suppression is used, multiple TIs are sampled, and the acquisition sequence includes pre-saturation. An *SI(PD)* map can be estimated by fitting a saturation recovery curve to a series of control images.

## 4. Technical Advances in ASL

The development of rs-fMRI was prompted by the consistent presence of intrinsic metabolic and perfusion patterns in the brain in the absence of a specific task. Resting-state blood oxygenation level dependent fMRI (rs-BOLD fMRI) and resting state ASL (rs-ASL) are the two main fMRI techniques [38].

### 4.1. Resting-State fMRI Using ASL

This technique allows functional connectivity measurements to be made for cognitive function and explains the psychological foundations of resting state connectivity.

Rs-fMRI using ASL (rs-fASL) has primarily been used for research purposes, but has also opened up new opportunities for pediatric neuroimaging [39].

Thus, investigating the properties of ASL perfusion during rest in the pediatric population may enhance our understanding of functional connectivity of the developing brain [40].

In rs-fASL acquisition, it is common practice to instruct participants to stay awake, keep their eyes open, not think about anything, and not fall asleep. 

The most commonly recommended fASL implementations are pCASL, owing to its simple implementation and relatively high SNR, and a 3D gradient and spin-echo (GRASE) readout, which has emerged as an alternative to the 2D-GE EPI readout, providing a higher SNR and more brain coverage. Hence, 3D GRASE acquisition in conjunction with pCASL and background suppression (BS) appears to be the current optimum protocol for dynamic rs-fASL [41].

Researchers recommend pre-processing rs-fASL data using MATLAB CONN toolbox, through the realignment of functional data, registration, and normalization [38]. Therefore, the post-processing step, which uses statistical parametric mapping software to investigate the temporal relationship between brain regions by extracting time series, is critical for investigating the rs-fASL technique [7].

Noninvasive rs-fASL is more convenient than task activation fMRI in infants and younger children, as it does not require the use of external tasks, as demonstrated by Liu et al. [7], who explored rs-ASL in developing human brains and examined the temporal relationship between the CBF of selected ROIs and that of other brain regions in young participants ranging in age from 6 to 20 years, in order to understand functional connectivity.

Moreover, quantified signal fluctuations of large-scale resting-state networks were studied by Dai et al. [42], by distinguishing between global signal fluctuations and signal fluctuations caused using random white noise. These authors demonstrated that network fluctuations become the dominant source of noise in rs-ASL CBF measurements at the lower spatial resolution typically introduced by smoothing and clustering analyses used for multi-participants studies.

### 4.2. BOLD fMRI and ASL

The ASL signal assesses the time course of a single physiological parameter and is not affected by scanner drifts because it does not depend on baseline changes [43]. It therefore improves the spatial specificity of neuronal activity, whereas rs-fMRI based on BOLD functional contrasts does not provide a direct and quantitative measure of brain function during rest and cannot be used in isolation to derive neuronal metabolism metrics [39].

Given the complexity of the BOLD signal, rs-ASL usefully complements BOLD fMRI [39].

In resting states, regional CBF could be used as a reasonable surrogate for metabolism. Thus, rs-fMRI using the ASL technique could improve the preoperative localization of the sensorimotor network, while BOLD has tremendous potential in clinical practice [44].

Moreover, BOLD fMRI data and ASL perfusion contrasts have been used to investigate the relationships between functional connectivity, regional CBF, and neurocognition in adult brains in resting and task states. It has been hypothesized that rs-BOLD fMRI measures are linked to regional CBF, which reflects underlying spontaneous brain activity [45,46]. In addition, cognitive decline could be detected before the emergence of clinical symptoms, in the form of disruption in regional CBF and impaired brain network connections.

To better explore ASL and rs-BOLD fMRI, the main acquisition parameters are summarized in Table 1.

Ultimately, comparable overall network connectivity reliability of ASL and BOLD was enhanced by collecting test-retest ASL MRI data in four resting-state task conditions and rs-BOLD fMRI [47].

## 5. Quality Control

### 5.1. Common Artifacts

Low SNR is a major issue in ASL imaging and can be exacerbated by artifacts [48]. As with any imaging examination, quality control is essential prior to any clinical assessment. Common artifacts, including motion, signal loss, distortion, bright spots, hyper/hypo-perfusion areas, and labeling failure, are described in Table 2 [14,22].

In addition, ASL should never be performed after administration of gadolinium-based contrast material, as the resulting T1 shortening is detrimental to the labeling.

### 5.2. Visual and Automated Quality Control

Several studies have focused on improving the quality of the image before any clinical investigation.

As mentioned earlier, ASL-CBF maps are susceptible to artifacts owing to a low SNR and motion sensitivity. ASL quality control is usually performed through the visual inspection of ASL-CBF maps, which is subjective, has potential for bias, and can be difficult to perform in a large sample size.

Quantitative signal targeting with alternating RF labeling of arterial regions (QUASAR) is one of these techniques introduced by Fallatah et al. [14]. QUASAR is a model-free ASL technique based on deconvolution with multi-slice/multi-TI data acquisition. A standardized image quality scoring (IQS) system has been developed to assess QUASAR ASL perfusion maps generated during clinical trials. It has two main components: (1) visibility vIQS and (2) the artifact aIQS. The sum of these two components yields the final IQS system score (IQS = vIQS + aIQS).

Likewise, Dolui et al. [49] devised a quality evaluation index for automatically assessing the objective quality of CBF maps. This index has a numerical value between 0 and 1, with higher values implying higher quality CBF maps. The factors considered are structural similarity, spatial variability, and negative CBF values in gray matter (GM).

Furthermore, the normalization of acquisition parameters and the correction of artifacts (movements, vascular artifacts, signal inhomogeneities, parallel imaging, and distortions) can be performed by the French Center for Image Acquisition and Processing (CATI). CATI is a national platform that facilitates the implementation of multicenter imaging studies to standardize acquisition protocols and perform quality assessment of the acquired images [50].

### 5.3. Quality Control of Negative Values in CBF Maps

Negative values in the ASL-CBF maps are frequently due to a low SNR. Thus, many studies have retained negative values, instead of excluding them, and, more precisely, have maintained volumes with a non-physiological negative mean GM-CBF [49,51].

Alternatively, thresholds can be applied in a quality control approach. Proisy et al. [52] worked on the qualitative analysis and quality control of CBF maps. They excluded negative values in CBF maps >20% in GM. A threshold was therefore set at 20% of negative GM values to be excluded.

Similarly, Wang [53] generated an image mask using a threshold of 20% of the maximum intensity of the mean perfusion image.

For their part, Bouhrara et al. [54] set the voxels with negative values to zero.

## 6. ASL-CBF Perfusion MRI Quantification

Recent studies have performed quantitative measures of CBF in healthy children and patients treated for pediatric tumors.

### 6.1. CBF in Healthy Children

The investigation of CBF over the course of childhood is key to understanding brain development, given its many age-related changes, both across the whole brain and in specific regions. In this context, Carsin-Vu et al. [16] studied CBF changes in GM, white matter (WM), the whole brain, and left and right hemispheres and lobes in 84 healthy children (44 girls/40 boys) aged between 6 months and 15 years (Table 3).

ASL-CBF measurements in GM tended to increase during the first years of life, with a peak at 3–4 years and a gradual decrease thereafter. In late adolescence, CBF seemed to show a small rebound. Given the close relationship between CBF and glucose consumption, the first increase in CBF could correspond to the overproduction of neurons and synapses during the first years of life and the second increase to the reduction in the number of synapses per neuron. Ultimately, there was no significant effect of sex on brain perfusion during childhood.

Additionally, measuring CBF in children allowed Yasuyuki Taki et al. [55] to establish a correlation between brain perfusion and age. The GM region of the cerebrum was divided into four lobes and 22 ROIs, each of which corresponded to anatomical structures in each hemisphere, using ROI analysis. The authors discovered that in most regions, the correlation between brain perfusion with GM density adjustment and age exhibited an inverted U-shaped trajectory followed by a U-shaped one. Furthermore, the age at which brain perfusion with GM density adjustment was the highest varied across the lobes and GM regions, and the association between brain perfusion with GM density adjustment and age increased from the occipital to the frontal lobe via the temporal and parietal lobes.

### 6.2. CBF in Pediatric Brain Tumors

Brain tumors are the most common solid tumors in childhood and the leading cause of cancer deaths in children.

ASL-CBF imaging is valuable in tumor diagnosis, surgical guidance, and therapeutic monitoring of brain tumors.

Recent research has shown that ASL is a reliable alternative to dynamic susceptibility contrast (DSC) and dynamic contrast-enhanced perfusion MRI techniques, which require contrast injection to assess tumor perfusion. ASL has distinct advantages in children: no need for contrast, efficient labeling, and potential for CBF quantification. ASL could also be used in cases of failed sedation or patient motion, which is a common problem in children with brain tumors [9].

#### 6.2.1. Classification of Tumor Types

It is essential to accurately identify brain tumors in children, in order to determine treatment strategies and prognoses. It is especially important to differentiate between medulloblastomas and pilocytic astrocytoma, as these are the two most common types of tumors found in the posterior fossa and have completely different therapeutic strategies and prognoses. Duc et al. [19] assessed ASL parameters for patients with medulloblastoma (*n* = 25) and patients with pilocytic astrocytoma (*n* = 8), quantifying ROI values for tumors. The median age of these two patient groups was 8 years for medulloblastoma and 6 years for pilocytic astrocytoma. According to World Health Organization (WHO) classification [56] medulloblastomas have greater malignancy than pilocytic astrocytoma, and CBF values were significantly higher for medulloblastomas than for pilocytic astrocytoma (Table 4).

#### 6.2.2. Tumor Grading

Disease severity in children varies greatly between tumor types, necessitating different approaches to early diagnosis. Dangouloff-Ros et al. [57] compared ASL data from low- and high-grade brain tumors in children without treatment to establish a cutoff for differentiating between low- and high-grade neoplasms and to assess potential correlations between CBF and quantitative histological microvascular data. ASL data were collected from 129 children between 2011 and 2015 and analyzed retrospectively. Table 5 sets out the results. High-grade pediatric brain tumors were found to have higher CBF than low-grade tumors, and these values could therefore be used to grade these tumors.

#### 6.2.3. CBF at Diagnosis

One potential benefit of using ASL-CBF as a non-invasive method is that brain tumors can be assessed at diagnosis to gauge their aggressiveness.

Vidyasagar et al. [17] measured CBF values in 23 pediatric patients (mean age: 8 years; range: 2–20 years; 11 girls and 12 boys) with brain tumors at first presentation and compared them with DSC values. No significant differences were found in ASL measurements between the tumor regions and control (healthy tissue) regions, suggesting that ASL is more sensitive than DSC measures to different physiological mechanisms (Table 6).

#### 6.2.4. CBF after Treatment

##### CBF in Children Treated for Ependymoma

Surgery and radiation therapy for pediatric tumors have been associated with declines in a variety of cognitive domains. The anatomical and physiological correlates of this cognitive decline are unknown, and no radiographic studies have been conducted on the long-term effects of this treatment paradigm.

CBF parameters would provide some insight into the structural and functional underpinnings of the cognitive differences observed in the pediatric population. In this context, Yecies et al. [20] conducted a comprehensive quantitative multimodal radiological analysis among seven long-term survivors of infratentorial ependymoma treated with surgery and conformal radiation (age at diagnosis: 4.2 years). There was a mean interval of 3.6 years between the end of treatment and follow-up imaging. The authors examined CBF in the supratentorial brain and compared the results with those of age matched, healthy children. They found several statistically significant differences in regional CBF in these patients compared with healthy controls. More specifically, they found decreased blood flow in the caudate and pallidum and increased blood flow in the nucleus accumbens (Table 7).

Long-term neurological sequelae have been found in children with sub-tentorial ependymoma treated via surgery and local radiotherapy, notably deficits in attention, reading, academic ability, and basic intelligence quotient.

This result in children who had undergone different treatment regimens suggests that changes in the tonsil may be related to the initial tumor and its surgical resection. The CBF changes detected in this study may be related to the cognitive deficits that have been identified in long-term survivors of ependymoma, as the nucleus accumbens plays an important role in the reward system. The caudate and pallidum are involved in motor and cognitive circuits. Modifications in any of these areas could potentially contribute to the academic difficulties and cognitive impairments seen in ependymoma survivors.

##### CBF in Children Treated for Pilocytic Astrocytoma, Glioblastoma, and Low-Grade Glioma

Novak et al. [18] looked into the relationships between MRI perfusion metrics measured via DSC and ASL in 15 patients with pediatric brain tumors (pilocytic astrocytoma (*n* = 7), glioblastoma (*n* = 1), and medulloblastoma *(n =* 1) who were treated with surgery and chemotherapy (or chemotherapy only). ASL-CBF measurements correlated well with DSC cerebral blood volume measurements in the tumors, and the correlation improved after leakage correction (Table 8). Signal normalization to unaffected tissue, such as GM or WM, may have improved ASL measurements, but it was difficult in some patients owing to large tumor size, which meant that there was little unaffected tissue within the ASL field of view.

##### CBF in Children Treated for Medulloblastoma

Many medulloblastoma survivors who have undergone surgery, radiotherapy, and chemotherapy experience long-term cognitive impairments and behavioral issues that can impair their quality of life. Processing speed, working memory, attention, and general intellectual ability deficits can result from the combined effect of disease burden and treatment toxicity. Matthew et al. [11] assessed the effects of treatment on the cerebral cortex, thalamus, caudate, putamen, globus pallidus, hippocampus, amygdala, nucleus accumbens, and WM, 5.7 years on average after the initial diagnosis in 21 patients with medulloblastoma aged 6.9–20.4 years (4 girls and 17 boys) and 64 healthy children. Patients treated with surgery, radiotherapy, and chemotherapy had a lower overall CBF than healthy children (Table 9).

The decrease in CBF observed in patients with medulloblastoma may be secondary to a reduction in microvascular density following irradiation. Chemotherapy could also be the cause of these changes, although there is a lack of published data examining CBF in the chronic phase after systemic chemotherapy only, without radiotherapy.

Moreover, Boisgontier et al. [58] used ASL perfusion imaging to investigate postoperative changes in whole-brain CBF at rest in 27 children with or without pediatric cerebellar mutism syndrome (pCMS) following medulloblastoma resection. Comparison of preoperative and postoperative ASL-CBF revealed a significant decrease in postoperative CBF in the left pre-supplementary motor area (pre-SMA) and SMA in patients with pCMS. There were no significant differences in patients who did not develop pCMS. Evidence that these areas are involved in speech production is consistent with the left pre-SMA and SMA hypoperfusion observed in children who developed mutism after MB resection.

## 7. ASL Perfusion Weighted Map Assessment

Several studies have undertaken qualitative and semi-quantitative assessments of ASL perfusion-weighted maps in pediatric patients.

### 7.1. Qualitative Assessment

Depending on the nature of the tumor being studied, and the nature of the treatment undergone, a qualitative assessment can be performed with the help of a neuroradiologist to reach a consensus on ASL map qualitative assessment.

ASL images can be scored on a 3 point scale (0 = definitely no increased perfusion compared with contralateral *“normal”* GM or WM, 1 = equivocal increased perfusion, 2 = definitely increased perfusion) [6,59].

This technique has been deemed inferior if the patient has poor contrast in a leaky region.

### 7.2. Semi-Quantitative Assessment in Pediatric Tumors

Semi-quantitative assessment in pediatric studies involves a health professional manually selecting an ROI in the region of the tumor judged to have a high perfusion value [60]. The ROI must then be normalized with a reference tissue in the patient’s brain to correct for age-dependent and patient-dependent variations in mean brain perfusion, such as the presence of hydrocephalus [61,62]. In ASL studies, normalization with contralateral GM has been recommended because of the higher SNR and shorter arterial transit time than in WM [6]. This ROI is sometimes positioned in a specific anatomical location, such as the contralateral thalamus [63] or temporal lobe [60]. However, in studies involving tumors in a variety of anatomical locations, a region of normal-appearing GM contralateral to each tumor is frequently used [6].

In this context, Hales et al. [63] used ASL MRI among healthy participants aged 8–32 years to look at changes in cerebral hemodynamics during normal development. They also investigated two patients aged 12–17 years. They extracted a tumor ROI, and a 150 mm^2^ ROI was placed in the normal GM.

Similarly, Morana et al. [60] compared the use of preoperative ASL and DSC MRI perfusion for tumor grading in pediatric patients with histologically proven, treatment-naïve low- or high-grade astrocytic tumors. They calculated the maximum value in the tumor ROI, divided by a similar ROI in contralateral GM in the temporal lobe.

Yeom et al. [6] also applied ASL perfusion to various pathological types of pediatric brain tumors that had no prior surgical resection, biopsy, or treatment and assessed the role of ASL in predicting tumor grade. They recorded the maximum ASL signal for each tumor ROI, and the two highest values were averaged. To correct for age-dependent and patient-dependent variations in mean brain perfusion, maximum tumor blood flow (TBF) was normalized to an ROI in contralateral GM (150 mm^2^) to produce the maximum relative TBF (rTBF).

In Table 10, we indicate the tumor type, age, location, and rTBF values.

Furthermore, Kikuchi et al. [64] assessed the relationship between TBF as measured via ASL and histopathological vascular density in treated pediatric brain tumors. They placed three to five ROIs (40–60 mm^2^) in the part of the tumor with maximum TBF. Three maximum ROI values were averaged for the TBF assessment. The maximum TBF was normalized to an ROI of 80 mm^2^ in contralateral GM to give a maximum relative TBF (rTBF) (Table 11).

Lastly, Testud et al. [65] explored the ability of ASL and DSC to distinguish between low- and high-grade lesions in an unselected cohort of children with newly diagnosed, treatment naïve intraparenchymal brain tumors. The ROI was placed on an ASL map, and parameters were normalized using an ROI in normal-appearing contralateral WM in the temporal lobe for supratentorial tumors and the cerebellar cortex for posterior fossa tumors.

## 8. Summary

ASL is a non-invasive imaging technique capable of studying brain tissue perfusion. It does not require the use of an exogenous contrast product, which is an advantage in the pediatric population, as it avoids the technical and ethical issues associated with the injection of contrast products.

It does, however, have several disadvantages as well. For example, owing to the low SNR, incoming labeled molecules only represent 1% of the static tissue signal, increasing the number of acquisitions needed, as well as the acquisition time, and even the risk of artifacts [53]. This means it is important to carry out quality control before embarking on a clinical investigation.

ASL-based CBF can be used as an alternative to the BOLD technique to measure resting-state functional connectivity. fMRI and ASL are also complementary, as they allow for a tight coupling between blood supply and the functional topology of the brain during rest and its modulation in response to an experimental paradigm, which may shed light on the physiological basis of the human brain’s functional connectome.

ASL-weighted perfusion maps and CBF maps are of interest for exploring, assessing, and quantifying brain tumors in pediatric patients before and after treatment. They should make it possible to limit the dose of irradiation to some structures and organs at risk in pediatric patients and by so doing limit the risks of cognitive sequelae after treatment [66]. Moreover, there are currently no established fluorescent stains that allow intraoperative imaging in pediatrics brain tumors. An ASL study was conducted by Lindner et al. [67] in healthy adults and glioblastoma patients. In a comparison with structural imaging, ASL was used both pre- and postoperatively to define tumor tissue and the extent of resection. The authors concluded that intraoperative ASL is a viable, reproducible, and reliable tool for mapping CBF in brain tumors and seems to provide more information than conventional intraoperative MR imaging in partial resection, which may be of interest in pediatric brain tumors.

## Figures and Tables

**Table 1:** Illustration of the different ASL and rs-BOLD fMRI acquisition parameters described in [46].

Acquisition Parameters	ASL Acquisition	Rs-BOLD fMRI
Acquisition type	CASL	echo-planar gradient
PLD	1000 ms	-
Labeling time	2000 ms	-
In-plane resolution	220 × 220 mm^2^	220 × 220 mm^2^
TE	17 ms	30 ms
TR	3800 ms	3000 ms
Flip angle	90°	90°
Slice thickness	7 mm	3 mm
Inter-slice gap	2.35 mm	-
Matrix	64 × 64 × 12	64 × 64
Image number	50 labeled/control image pairs	220 images

**Table 2:** Common artifacts and their definitions.

Common Artifacts	Definitions
Motion artifacts	Appear as rings or curved lines and can cause artificially high or low CBF values.
Signal loss	Results from susceptibility effects with EPI-based playback sequences. These typically occur at air-tissue interfaces, such as near the frontal sinuses or the mastoid bone.
Distortions	
Bright spots (or macrovascular artifacts)	Are random clusters of very high perfusion voxels caused by the residual vascular signal.
Hyper/Hypo-perfusion	Is sometimes visible on perfusion maps, without being either a pathological or acquisition artifact. This constitutes a physiological change in perfusion.
Labeling failure	Failure to label incoming blood due to local susceptibility artifacts results in an apparent lack of perfusion throughout the affected vascular territory.

**Table 3:** Absolute CBF values (mL/100 g/min) for each age group for the whole brain, gray matter (GM) and white matter (WM) [16].

Imaging Technique: 3D Pulsed ASL on 1.5T MAGNETOM Aera			
Age	Whole Brain CBF	CBF in GM	CBF in WM
6–11 months	53.3 ± 7.8	58.6 ± 8.3	29.2 ± 5.1
12–23 months	61.7 ± 3.2	68.2 ± 3.5	39.3 ± 2.5
2–3 years	68.5 ± 4.4	76.5 ± 4.9	40.2 ± 4.5
4–5 years	56.6 ± 3.8	64.9 ± 4.3	26.0 ± 2.1
6–7 years	62.4 ± 3.0	71.4 ± 3.1	30.5 ± 2.3
8–9 years	54.9 ± 2.7	63.9 ± 3.1	25.8 ± 1.8
10–11 years	53.4 ± 5.2	62.4 ± 6.1	23.9 ± 2.6
12–13 years	43.3 ± 2.6	51.0 ± 3.0	21.7 ± 2.0
14–15 years	50.1 ± 2.0	59.3 ± 2.5	24.8 ± 1.2

**Table 4:** Absolute CBF values (mL/100 g/min) for each tumor ROI [19].

Imaging Technique: Axial 3D Pseudo Continuous ASL with 16-Channel Head Coil in a 1.5 T MRI Machine	
Tumor Type	CBF
Medulloblastoma	16.02
Pilocytic astrocytoma	9.28

**Table 5:** Absolute CBF values (mL/100/min) according to age, brain structure, and tumor [57].

Imaging Technique: 3D Pseudo Continuous ASL MR Imaging on a GE Signa HDxt 1.5 T System with a 12-Channel Head-Neck-Spine Coil			
Age in Years	Brain Structure	Tumor Type	CBF without Treatment
2.3–9.5	Posterior fossa	Pilocytic astrocytoma	32 (25–40)
4.6–10.5		Medulloblastoma	59 (48–87)
1.2–2.6		Grade 3 ependymoma	82 (47–142)
2.7–5.6	Thalamus	Pilocytic astrocytoma	36 (30–40)
8.9–14.2		Grade 3 astrocytoma	73 (64–241)
6.6–7.4		Glioblastoma	94 (90–97)
4.9–12.7	Hemispheres	Glioblastoma	117 (100–130)

**Table 6:** Absolute CBF values (mL/100 g/min) within both tumor and healthy tissue regions of interest [17].

Imaging Technique: Spin Tagging with Alternating RF Labeling Scheme (STAR) with a Look–Locker Readout on a 3T Philips Achieva		
	Tumor Type	CBF
CBF in control regions	-	75
CBF in tumor regions	Low-grade glioma	87
	Medulloblastoma	111

**Table 7:** *p* values for median CBF in ependymoma survivors versus healthy children of the same age [20].

Imaging Technique: 3D Pseudo Continuous Labeling on a 3 T MRI Scanner	
Brain Structure	*p* Value
White matter	0.749
Cerebral cortex	0.742
Thalamus	0.650
Caudate	**0.050**
Putamen	0.124
Globus pallidus	**0.029**

Bold: The statistically significant differences in regional CBF in these brain structures compared with healthy controls.

**Table 8:** Absolute CBF values (mL/100 g/min) [18].

Imaging Technique: Pseudo-Continuous ASL Sequence on a Philips Achieva 3T TX System (Best, The Netherlands) Using a 32-Channel Head Coil			
Age (Years)	Brain Structure	Tumor Type	Absolute CBF Value
11.3	Thalamus	Pilocytic astrocytoma	79.49
4.8	Optic chiasm	Pilocytic astrocytoma	86.12
3.9	Optic chiasm	Pilocytic astrocytoma	56.03
2.8	Optic chiasm	Pilocytic astrocytoma	52.50 (chemotherapy only)
5.6	Optic pathway	Pilocytic astrocytoma	48.21 (chemotherapy only)
2.1	Left hemisphere	Glioblastoma	10.94
5.3	Optic pathway	Low grade glioma	82.62 (chemotherapy only)

**Table 9:** Absolute CBF values (mL/100 g/min) in patients treated for medulloblastomas and neurologically normal children [11].

Imaging Technique: 3D Pseudo-Continuous Labeling on a 3T MRI Scanner		
Brain Structure	Absolute CBF Values in Healthy Controls	Absolute CBF Values in Patients with Medulloblastoma
White matter	45.6–49.2	37.9–44.4
Cerebral cortex	64.2–69.7	47.8–57.9
Thalamus	53.4–58.2	38.6–47.3
Caudate	53.1–56.8	43.8–50.6
Putamen	54.9–58.6	44.6–51.4
Globus pallidus	42.3–45.9	32.9–39.4
Hippocampus	52.2–56.5	41.1–49
Amygdala	49–53.2	37.4–44.9
Nucleus accumbens	56.5–60.6	49.1–56.7

**Table 10:** Relative tumor blood flow (rTBF) values in different pediatric tumors, age of patients, and tumor location [6].

Tumor Type	Mean Age (Years)	Tumor Location	rTBF
Glioblastoma	13 ± 8.5	Cerebrum	3.70 ± 1.89
Anaplastic astrocytoma	11	Cerebrum	3.60
Medulloblastoma	6.1 ± 3.7	Posterior fossa	2.87 ± 1.74
Pilocytic astrocytoma	9.4 ± 6.1	Cerebrum, Cerebellum, Brainstem	1.05 ± 0.19
Ependymoma	3	Posterior fossa	1.82

**Table 11:** rTBF values for different pediatric tumors according to age [64].

Histopathology	Age (Years)	Sex	rTBF
Pilocytic astrocytoma	3	Male	0.54
Pilocytic astrocytoma	3	Female	0.29
Pilocytic astrocytoma	6	Male	0.36
Pilocytic astrocytoma	11	Female	0.53
Glioblastomas	9	Female	0.78
Anaplastic ependymoma	2	Female	2.00
Anaplastic ependymoma	3	Male	1.44
Anaplastic ependymoma	6	Female	1.98
Medulloblastoma	9	Male	3.59
Medulloblastoma	11	Male	0.82
